# Lifespan Disparity as an Additional Indicator for Evaluating Mortality Forecasts

**DOI:** 10.1007/s13524-017-0584-0

**Published:** 2017-07-05

**Authors:** Christina Bohk-Ewald, Marcus Ebeling, Roland Rau

**Affiliations:** 1 Max Planck Institute for Demographic Research, Konrad Zuse Strasse 1, 18057 Rostock, Germany; 2 Chair for Demography, University of Rostock, Ulmenstrasse 1, 18057 Rostock, Germany; 3 Max Planck Research Center on the Biodemography of Aging, Department of Public Health, University of Southern Denmark, Campusvej 55, 5230 Odense, Denmark

**Keywords:** Evaluation, Forecasting performance, Lifespan disparity, Average lifespan

## Abstract

Evaluating the predictive ability of mortality forecasts is important yet difficult. Death rates and mean lifespan are basic life table functions typically used to analyze to what extent the forecasts deviate from their realized values. Although these parameters are useful for specifying precisely how mortality has been forecasted, they cannot be used to assess whether the underlying mortality developments are plausible. We therefore propose that in addition to looking at average lifespan, we should examine whether the forecasted variability of the age at death is a plausible continuation of past trends. The validation of mortality forecasts for Italy, Japan, and Denmark demonstrates that their predictive performance can be evaluated more comprehensively by analyzing both the average lifespan and lifespan disparity—that is, by jointly analyzing the mean and the dispersion of mortality. Approaches that account for dynamic age shifts in survival improvements appear to perform better than others that enforce relatively invariant patterns. However, because forecasting approaches are designed to capture trends in average mortality, we argue that studying lifespan disparity may also help to improve the methodology and thus the predictive ability of mortality forecasts.

**Electronic supplementary material:**

The online version of this article (doi:10.1007/s13524-017-0584-0) contains supplementary material, which is available to authorized users.

## Introduction

The aim of most mortality forecasts is to predict how many additional years of life people will gain in the future. Basic life table functions—such as life expectancy at birth (a measure of central tendency) and age-specific death rates (measures of mortality intensity) are usually applied to evaluate the precision of such forecasts. The closer a forecast is to the observed development, the greater is its forecasting performance—or, interchangeably, its predictive ability. Goodness-of-fit tests as well as validation procedures are typically used to evaluate the predictive ability of mortality forecasts. Placing particular emphasis on *ex post* quantitative aspects (Armstrong and Collopy 1992; Cairns et al. 2011b; Keilman 1997; Shang 2015), conventional evaluation measures quantify the difference between predicted and observed mortality. It is commonly considered that the greater such forecast errors, the poorer is the forecasting performance. However, although deviations are supposed to be small, zero deviations would indicate overfitting rather than a good forecasting performance. Forecast errors can be expressed in absolute or relative terms, and they can be averaged over dimensions such as age, time, and population (Booth et al. 2006; Keilman and Pham 2004; Koissi et al. 2006; Shang et al. 2011; Smith et al. 2001). The meaning of these errors changes in each case. For example, means of absolute errors measure accuracy, whereas means of positive and negative errors measure bias—that is, systematic over- or underestimation. Relative errors deal with scale dependency and therefore allow comparison of errors across measures and methods. Dowd et al. (2010); Koissi et al. (2006), and Lee and Miller (2001) analyzed how errors or (standardized) residuals are distributed. In addition to employing visualization techniques, these authors used statistical tests such as chi-squared, Levene’s test, the variance ratio test, or the Jarque-Bera normality test. Moreover, Shang (2015) recently proposed using test statistics to reveal significant differences in the forecast accuracy of point and interval estimates as well as differences between the forecasts of multiple approaches.

**Fig. 1:**
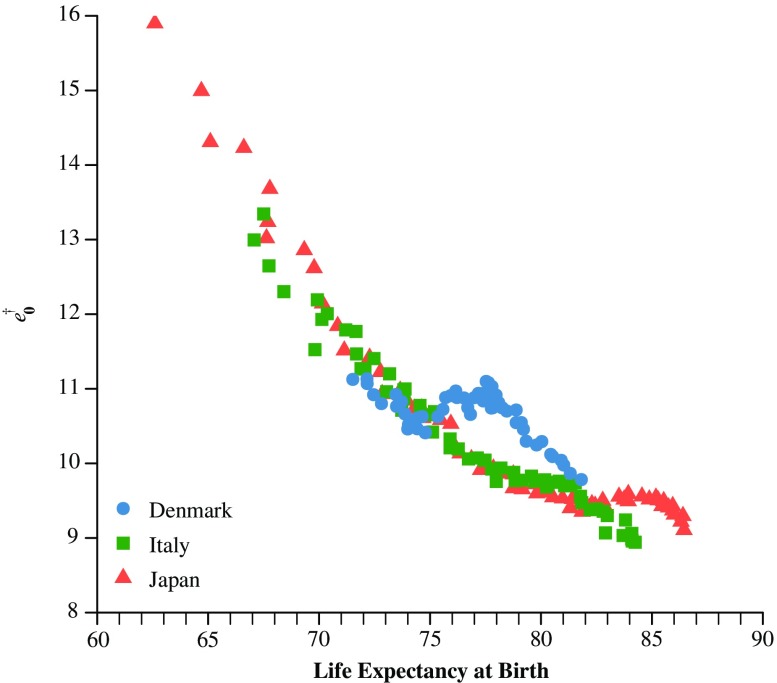
Scatterplot of life expectancy at birth and average life years lost at birth due to death for women in Denmark, Italy, and Japan from 1950 to 2012

Errors and test statistics of basic life table functions are useful for specifying precisely how mortality has been forecasted. However, small errors in the forecasts of average lifespan do not necessarily indicate that the forecasted underlying mortality developments are plausible. Figure 1 illustrates this issue in more detail with a scatterplot that displays the negative correlation between life expectancy at birth and lifespan disparity measured by average life years lost at birth, ${e}_0^{\dagger }$ (e.g., Vaupel and Canudas-Romo 2003), for women in Italy, Denmark, and Japan from 1950 to 2012. In contrast to basic life table measures, ${e}_0^{\dagger }$ provides information about the underlying mortality developments. Although life expectancy at birth has increased in recent decades because of reductions in mortality at progressively higher ages, ${e}_0^{\dagger }$ has decreased mainly as a result of survival improvements at premature ages, which shifted deaths toward the end of the lifespan. Figure 1 shows a striking pattern: the average lifespan of Italian, Danish, and Japanese women has been similar in recent decades, whereas the decline in the variability of the age at death differed considerably among these groups of women as soon as their average lifespan exceeded 75 years. Specifically, lifespan dispersion (1) declined regularly for Italian women, (2) leveled off for Japanese women, and (3) increased and decreased for Danish women.

These findings illustrate that different underlying mortality trajectories can lead to similar average lifespans and lifespan disparities. Other researchers have discussed this relationship in detail (e.g., Smits and Monden 2009; Vaupel et al. 2011; Wilmoth and Horiuchi 1999). For example, Wilmoth and Horiuchi (1999) showed that different levels of life expectancy at birth can come across with different levels of lifespan dispersion. Goldstein and Cassidy (2012) and Bergeron-Boucher et al. (2015) analyzed the impact of changing slopes in the mortality age profile on life expectancy at birth and lifespan dispersion. For instance, Goldstein and Cassidy concluded that changes in the slope have a relatively larger effect on life expectancy at birth than changes in the level of mortality. To ensure that we take these underlying trajectories into account, we propose expanding the toolkit of conventional evaluation procedures. Basic life table functions should be complemented by measures of lifespan dispersion to improve the assessment of *ex post* quantitative aspects and to evaluate the plausibility of underlying mortality trends. To the best of our knowledge, only Cairns et al. (2006, 2011b) have taken a similar approach. They added an *ex ante* evaluation with qualitative criteria to *ex post* measures; that is, they examined the forecasting performance using qualitative criteria, such as the biological validity of the age schedule of mortality, and investigated the consistency of the forecasts using historical data. However, as far as we know, no existing studies have used lifespan disparity as an evaluation measure for the plausibility of mortality forecasts. The objective of this article is to highlight the necessity to assess whether mortality forecasting methods can capture and forecast different trends of life expectancy at birth and lifespan disparity—that is, expose the benefits of incorporating lifespan disparity as an additional indicator in the toolkit that is used to evaluate the performance of mortality forecasts.

## Lifespan Disparity: Measures and Concepts

*Lifespan disparity* describes the variation in the lifespan distribution—that is, the differences in the length of life across members of a population. A wide range of approaches can be used to measure lifespan disparity, including (1) classic statistical variability measures, such as the standard deviation or the interquartile range; (2) equality measures, such as the Gini coefficient; or (3) geometric approaches, such as the Prolate index (Cheung et al. 2005; Eakin and Witten 1995; Kannisto 2000; Wilmoth and Horiuchi 1999). However, because all these measures are highly correlated (Vaupel et al. 2011; Wilmoth and Horiuchi 1999), we can expect that their impact on the results would be minor. Although those measures are highly correlated, however, their trends may differ. For example, if equality were rising, measures of variability would decrease, whereas measures of rectangularity would increase (Wilmoth and Horiuchi 1999).

To measure lifespan dispersion,^1^ we take the average number of life years lost at birth (Vaupel and Canudas-Romo 2003; Zhang and Vaupel 2009), ${e}_0^{\dagger }$, estimated by

1 $$ {e}_0^{\dagger }=\frac{\underset{0}{\overset{\upomega}{\int }}{e}_a{d}_a da}{l_0}, $$

with *e*_*a*_ being remaining life expectancy at age *a*, and *d*_*a*_ being life table deaths at age *a*, with both integrated from age 0 to ω, the highest age at death. *l*_0_ is the radix of the life table. A major reason why we chose ${e}_0^{\dagger }$ is that it is demographically interpretable as the average life years lost. Because ${e}_0^{\dagger }$ refers to the lost living potential, it also provides information about the capacity for further increases in life expectancy. We argue that these key features enable ${e}_0^{\dagger }$ in particular to be used to evaluate the plausibility of mortality forecasts.

Measuring lifespan disparity may reveal one of three general patterns: the compression, shifting, or expansion of mortality. Although these patterns are not mutually exclusive in the real world, they are useful for explaining trends in lifespan disparity. Fries (1980) established the concept of mortality compression, originally describing a postponement of mortality to some fixed upper lifespan limit, which in turn induces a reduction in lifespan disparity. Although the expected levels of lifespan disparity have not been reached and the proposed levels of average lifespan have been exceeded, the concept of mortality compression is typically used to describe the massive reductions in lifespan variability since the mid-nineteenth century (Kannisto 2000; Nagnur 1986; Nusselder and Mackenbach 1996). The concept of shifting mortality describes a postponement of the old-age death bulk toward higher ages with an approximately constant level of lifespan variability. Empirical studies have provided evidence that shifting mortality may occur following mortality compression (e.g., Bongaarts 2005; Canudas-Romo 2008; Kannisto 1996). The concept of mortality expansion refers to progressive improvements in survival to very old ages that have not previously been reached by many people. Mortality expansion to very old ages induces temporarily increasing lifespan variability, although its impact on total variability of the age at death has not been evident until recently. However, increasing lifespan dispersion has been observed in multiple populations at approximately age 60 (Engelman et al. 2010, 2014; Rothenberg et al. 1991).

A (positive or negative) change in life expectancy at birth, along with a compression, a shifting, and/or an expansion of mortality, are possible developments that should be captured in a mortality forecast. Because these developments are closely related and occur at different times in different populations, mortality forecasting approaches may need to be adjusted to ensure that they are captured appropriately.

## Mortality Forecasting Approaches That Tackle Variability of the Age at Death

### Concise Overview

Many approaches, like the canonical Lee-Carter model (1992), extrapolate past trends while assuming that the relative progress in mortality made among people of different ages has been time-invariant. This assumption is, however, implausible, given that survival improvements differ considerably by age over time. In the first half of the twentieth century, large reductions in mortality occurred among infants and young children in many highly developed countries. More recently, most of the survival improvements have been among adults and the elderly. In the coming decades, mortality is expected to decline mainly among the very old. Hence, the assumption of time-invariant changes in mortality by age may induce forecasts that are prone to errors. Recently developed approaches respond to this problem in different ways. For example, Janssen and de Beer (2016) accounted for the distribution of the age at death; Li et al. (2013) rotated the age pattern of mortality change with time; and Haberman and Renshaw (2012), Mitchell et al. (2013), and Bohk-Ewald and Rau (2017) used rates of mortality improvement instead of death rates to forecast dynamic age shifts in mortality decline. Moreover, Li and Lee (2005), Cairns et al. (2011a), Hyndman et al. (2013), and others used coherent approaches to jointly forecast mortality among multiple populations, allowing populations to adapt their below- or above-average increases in life expectancy to a shared trend among multiple populations. Capturing ruptures in long-term trends that emerge from irregular patterns of mortality change is also challenging. For example, Coelho and Nunes (2011) dealt with long-term trend changes in mortality forecasts, Janssen et al. (2013) included exogenous variables such as tobacco smoking, and Renshaw and Haberman (2006) considered cohort mortality to account for this issue.

We select three of these approaches, which differ in their ability to capture dynamic age shifts in survival improvement, to forecast mortality exemplarily for women in Italy, Japan, and Denmark up to 2009 (see the next section). These models are the Lee-Carter model, its rotating variant developed by Li et al., and the model developed by Bohk-Ewald and Rau. Given that their levels of modeling flexibility differ, each approach models the various trends in lifespan disparity in the three populations differently (see Fig. 1). All the approaches mentioned in the concise overview are equally qualified to be selected for the case studies to show the advantages when evaluating the forecasting performance using the mean—and, as an extra criterion, the spread of mortality. Hence, this analysis is designed to show the additional information that can be gained when evaluating the forecasted spread of mortality in the presence of different trends for life expectancy at birth and lifespan dispersion. Although the case studies provide some results for comparing the forecasting performance of the three approaches, this should be considered preliminary and rather a byproduct than an incentive to conduct this analysis; a valid model comparison would instead require a systematic evaluation of the forecasting performance using extensive mortality data of multiple countries and periods, which is beyond the scope of this work. Hence, for the case studies, we select three forecasting models that cover the range of available approaches and modeling strategies quite well. In addition to describing the method-based assumptions of the selected approaches for capturing dynamic age shifts in survival improvement, as well as some details on implementation, we offer hypotheses regarding the effect that each approach might have on the forecasted mean lifespan and lifespan disparity.

### Impact of Model-Based Assumptions on Lifespan Disparity

#### Lee-Carter Model

Although the Lee-Carter model has been used and revised extensively since it was first developed in 1992 (Booth and Tickle 2008; Booth et al. 2006; Butt and Haberman 2010; Shang 2012; Shang et al. 2011), we use its original version as a benchmark in our case studies. The Lee-Carter model forecasts mortality by age and calendar year on the logarithmic scale while assuming that the relative changes in mortality were constant between the ages over time. Hence, if the survival improvements were relatively large at young ages and small at old ages in the reference years, this proportion would assumedly be unchanged in the forecast years. Yet, given the shifts by age in survival improvements over time, we hypothesize that the inflexibility in the age profile of mortality change would have affected the Lee-Carter forecasts up to 2009. The extrapolation of declining mortality at infant, child, adult, and old ages based solely on the mortality trends observed in the reference period may result in a reliable forecast for the near future given that the prevalence of mortality reductions at very old ages will still be low. However, if mortality continues to decline at progressively higher ages in the coming decades, the Lee-Carter model may produce forecasts that fail to capture correctly both the average lifespan and lifespan disparity. The absence of a dynamic shift in survival improvements to progressively higher ages may then induce (1) an underestimation of life expectancy at birth as well as (2) a strong compression of deaths, which may in turn be accompanied by a strong decline in the lifespan dispersion. To generate the mortality forecasts with the Lee-Carter model, we implemented the model in the statistical software R (R Core Team 2015).

#### Li et al. Model

Many scholars have refined the Lee-Carter model to address the problem of the inflexibility in the age profile of mortality change (Booth et al. 2006; Shang et al. 2011; Soneji and King 2011). Li et al. (2013) took an important step in this direction by implementing a time-variant age schedule of mortality change that rotates from a present level to an ultimate level. The timing and the pace of the rotation depend on the average lifespan, which has been forecasted in a previous step with the original Lee-Carter model. As soon as life expectancy at birth exceeds a value of 80 years, the rotation starts; it then proceeds until life expectancy at birth reaches an ultimate level of 102 years. The greater the number of forecasted additional years of life is, the faster the ultimate schedule is achieved, and the rotation stops. The ultimate schedule of mortality change is constant for ages 0 to 64, and it gradually declines thereafter. The rotation basically induces a postponement of relatively large survival improvements from younger to older ages. Given that the average lifespan is forecasted using the original Lee-Carter model, the rotation affects only the underlying mortality dynamics—not the average level of mortality. Assuming a regular decline in mortality, we expect to find that (1) like the original model, the rotated model may underestimate additional years of life; but (2) unlike the original model, it may be able to forecast a mortality compression that is less strong because of its greater modeling flexibility. To take these dynamic mortality changes into account, Ševčíková et al. (2016) implemented the rotation in Raftery et al.’s (2013) model, which has been used in the UN World Population Prospects (2014, 2015). To derive the age profiles of mortality using the rotated Lee-Carter model, we implement this model in R with a few adjustments. Because we allow approaches to shift deaths beyond the maximum age of the data (see the upcoming section, “Estimation and Evaluation Procedure”), we set the ultimate schedule of mortality change constant until age 80, and as gradually declining thereafter. Moreover, for the rotation, we change the recommended bounds of the forecasted lifespan, which are 80 years and 102 years. We set the lower bound at 75 because differences in lifespan disparity started to develop for women in Italy, Japan, and Denmark as the average lifespan exceeded this value (see Fig. 1). Finally, to avoid jump-off bias, we use the last observed death rates to forecast mortality.

#### Bohk and Rau Model

The model of Bohk-Ewald and Rau (2017) provides an alternative strategy for forecasting that relatively large rates of mortality improvement proceed from younger to older ages. This model predicts survival improvements instead of death rates, and it optionally combines the mortality trends of multiple populations to account for (anticipated) trend changes in the forecast years. Although this model allows us to assume mortality convergence between a country of interest and reference countries, we do not use this feature in the case studies in order to enable a fair comparison with the model of Li et al. (2013). Moreover, the Bohk and Rau model has a linear and an exponential core model to forecast time series of age-specific mortality change, using simulation-based Bayesian inference to run those models and to estimate coherent changes of mortality among adjacent ages. The model has been applied to forecast mortality for some European countries (Bohk and Rau 2014; Bohk-Ewald and Rau 2017) as well as for the United States (Bohk and Rau 2016). Although both the rotating Lee-Carter model and the Bohk and Rau model allow the age profile of the rates of mortality improvement to change, the latter model appears to be more flexible because it does not assume an approximation of an ultimate schedule. If mortality declines regularly, we expect that the Bohk and Rau model will perform as well as the rotating Lee-Carter model in forecasting average mortality and lifespan disparity and that it will perform even better in generating forecasts for populations with irregular mortality developments because it is more adaptable to different forecasting situations. To generate the forecasts with the Bohk and Rau model, we use its implementation in R, which is described in detail in Bohk-Ewald and Rau (2017).

## Illustrative Examples

In this section, we validate the forecasting performance of the Lee-Carter model, its rotating variant, and the model of Bohk-Ewald and Rau. Using illustrative examples, we examine whether each model is able to generate precise forecasts of average mortality and lifespan disparity. These illustrative examples are designed to indicate whether the approaches can capture (1) regular and irregular trends of average lifespan and (2) dynamic age shifts in survival improvements.

### Estimation and Evaluation Procedure

The mortality forecasts up to 2009 rely on four reference periods (1965–1990, 1960–1985, 1955–1980, and 1950–1975). We compare the estimations with the observed values. Besides *e*_0_, which is a common indicator in evaluations, we also compare the forecasted ${e}_0^{\dagger }$ values with the observed values to assess the ability of the forecasting approaches to predict average mortality and lifespan disparity. We focus our main analysis on *e*_0_ and ${e}_0^{\dagger }$, but we also provide results for *e*_65_ and ${e}_{65}^{\dagger }$ in Online Resource 1 in order to show how sensitive (or robust) our findings are.

We employ visualization techniques as well as forecast errors to evaluate the forecasting performance of each method. To quantify forecast accuracy in terms of the mean and spread of mortality, we use the absolute percentage error (APE) because it is a relative error that relates the absolute difference between forecasted and observed values to the size of the actual values. Because the APE can, therefore, deal with measures of different scales, we use it to compare the forecasting performance (across time and by country) between the methods using *e*_0_ and ${e}_0^{\dagger }$. Given that the chosen approaches provide probabilistic mortality forecasts, we focus not only on the evaluation of median point estimates but also on the calibration of prediction intervals. We use empirical frequencies to evaluate the uncertainty estimates of probabilistic forecasts; empirical frequencies give the proportion of observed values that actually fall within the prediction intervals. For instance, a 95 % prediction interval should capture 95 % of all observations. If it captures more or fewer observations, it is too wide or too narrow, respectively (e.g., Raftery et al. 2013; Schmertmann et al. 2014).

We generate forecasts of mortality for women in Italy (regular *e*_0_ and ${e}_0^{\dagger }$), Japan (regular *e*_0_ and irregular ${e}_0^{\dagger }$), and Denmark (irregular *e*_0_ and ${e}_0^{\dagger }$) because these groups have differed substantially in recent decades in their levels of life expectancy and lifespan dispersion (see Fig. 1). As input data, we use deaths and exposures by single age from 0 to 110+, and by calendar year from 1950 to 2009, from the Human Mortality Database (n.d.). To enable the forecasting approaches to shift deaths to ages beyond 110+, we extend the age range to 130+ with the Kannisto model (Thatcher et al. 1998), the details of which we explain in Online Resource 1 (section A). This approach is similar to Ševčíková et al.’s (2016) revised UN approach. The estimation of *e*_0_ and ${e}_0^{\dagger }$ (and of *e*_65_ and ${e}_{65}^{\dagger }$) is based on life tables produced from the forecasted and observed age-specific death rates.

### Results

**Fig. 2:**
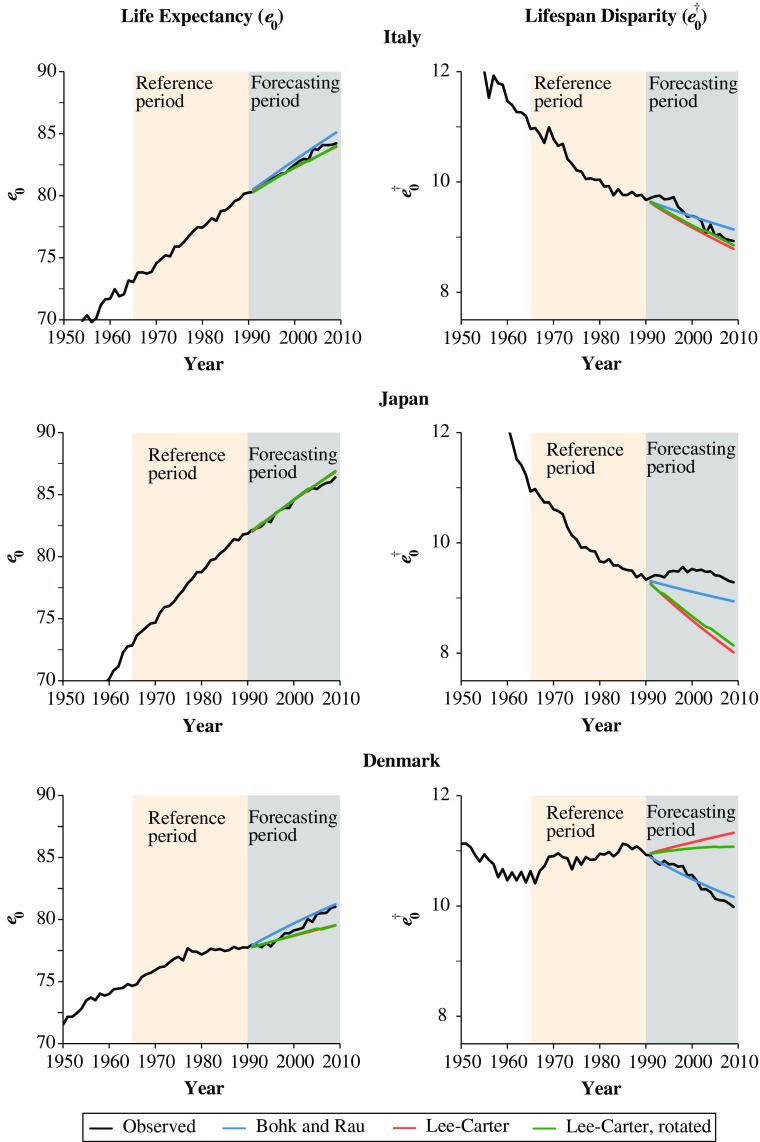
Life expectancy at birth (left panels) and life years lost at birth (right panels) for women in Italy, Japan, and Denmark: Observed data, forecasted data using the Lee-Carter model, the Lee-Carter rotating variant (proposed by Li et al. 2013), and the Bohk and Rau model. Forecast years are 1991–2009. Reference period is 1965–1990

*Visualize Forecast Performance* Figure 2 displays the average lifespan, *e*_0_, and the average number of life years lost, ${e}_0^{\dagger }$, for women in Italy, Japan, and Denmark. The observed data are in black, and the forecasted data are in red (Lee-Carter model), green (rotating variant proposed by Li et al. 2013), and blue (Bohk and Rau model). Moreover, the forecasted years (1991–2009) are highlighted in gray, and the reference period (1965–1990), is highlighted in beige. Given the technical construction of the Lee-Carter models, it is not surprising that the forecasts of average lifespan are almost identical: both models use the forecasted life expectancy at birth of the original model. The rotated variant deviates no more than +/– 0.1 years, which we used as a tolerance level when adjusting the age profile of mortality change with the rotation to fit the average lifespan of the original Lee-Carter model. By contrast, the forecasts of the Lee-Carter models differ in terms of lifespan disparity. The effect shown here is greater than it would have been with Li et al.’s (2013) original implementation because we let the rotation start when the average lifespan exceeded the value of 75 years, not of 80 years.

*Quantify Forecast Performance* Table 1 lists the mean of the APEs for *e*_0_ and ${e}_0^{\dagger }$ over the forecast years by country and forecasting method for each validation setting, and Table 2 lists those mean absolute percentage errors (MAPEs) averaged over all four validation settings. Strikingly, the MAPEs appear to be greater for ${e}_0^{\dagger }$ than for *e*_0_ for almost any country, validation setting, and method. The overall mean of all MAPEs is approximately 0.01 for *e*_0_ and 0.046 for ${e}_0^{\dagger }$; that is, the forecasts deviate on average by 1 % from life expectancy at birth and by 4.6 % from lifespan dispersion. As a consequence, the forecasting performance is depreciated for all methods when we consider ${e}_0^{\dagger }$ in addition to *e*_0_. Furthermore, the MAPEs for *e*_65_ and ${e}_{65}^{\dagger }$ are listed in Tables S1 and S2 in Online Resource 1. In contrast with mortality over the entire lifespan, errors often appear to be smaller for ${e}_{65}^{\dagger }$ than for *e*_65_. An exception is Japan; in the validation settings 1 and 2, the errors appear to be larger for ${e}_{65}^{\dagger }$ than for *e*_65_.

**Table 1:** Mean of the absolute percentage errors (MAPE) for *e*_0_ and ${e}_0^{\dagger }$ over the forecast years by country and method

Country and Measure	Method		
	Lee-Carter	Lee-Carter, Rotated (Li et al.)	Bohk and Rau
Validation 1 (ref. years: 1965–1990; forecast years: 1991–2009)			
Italy			
*e*_0_	0.003	0.003	0.005
${e}_0^{\dagger }$	0.019	0.015	0.014
Japan			
*e*_0_	0.002	0.003	0.002
${e}_0^{\dagger }$	0.087	0.080	0.034
Denmark			
*e*_0_	0.008	0.007	0.006
${e}_0^{\dagger }$	0.065	0.054	0.009
Validation 2 (ref. years: 1960–1985; forecast years: 1986–2009)			
Italy			
*e*_0_	0.010	0.010	0.002
${e}_0^{\dagger }$	0.029	0.021	0.018
Japan			
*e*_0_	0.002	0.002	0.004
${e}_0^{\dagger }$	0.092	0.076	0.014
Denmark			
*e*_0_	0.005	0.004	0.017
${e}_0^{\dagger }$	0.094	0.077	0.021
Validation 3 (ref. years: 1955–1980; forecast years: 1981–2009)			
Italy			
*e*_0_	0.014	0.014	0.002
${e}_0^{\dagger }$	0.027	0.019	0.012
Japan			
*e*_0_	0.009	0.009	0.003
${e}_0^{\dagger }$	0.118	0.092	0.022
Denmark			
*e*_0_	0.007	0.008	0.029
${e}_0^{\dagger }$	0.048	0.033	0.023
Validation 4 (ref. years: 1950–1975; forecast years: 1976–2009)			
Italy			
*e*_0_	0.018	0.018	0.008
${e}_0^{\dagger }$	0.032	0.023	0.053
Japan			
*e*_0_	0.018	0.018	0.012
${e}_0^{\dagger }$	0.131	0.094	0.032
Denmark			
*e*_0_	0.015	0.014	0.035
${e}_0^{\dagger }$	0.020	0.018	0.043

*Note:* MAPEs are shown for four validating settings that all forecast mortality until 2009, but they use different historical periods.

**Table 2:** Mean of the absolute percentage errors (MAPE) for *e*_0_ and ${e}_0^{\dagger }$ over all validation settings by country and method

Country and Measure	Method		
	Lee-Carter	Lee-Carter, Rotated (Li et al.)	Bohk and Rau
Italy			
*e*_0_	0.011	0.011	0.004
${e}_0^{\dagger }$	0.027	0.019	0.024
Japan			
*e*_0_	0.008	0.008	0.005
${e}_0^{\dagger }$	0.107	0.086	0.025
Denmark			
*e*_0_	0.009	0.008	0.022
${e}_0^{\dagger }$	0.057	0.045	0.024

The empirical frequencies for *e*_0_ and ${e}_0^{\dagger }$ in Table S3 and for *e*_65_ and ${e}_{65}^{\dagger }$ in Table S4 (Online Resource 1) confirm our findings for the median forecasts and show even more clearly that current approaches struggle to forecast lifespan disparity. The 95 % prediction intervals capture, on average, a fairly large number of observations for life expectancy at birth and, albeit slightly fewer, for remaining life expectancy at age 65. By contrast, many fewer observations are captured by the 95 % prediction intervals for lifespan disparity; empirical frequencies range from 0 % to 96.5 %, with the average being only approximately 26 %.

#### Case of Italy: Regular Trends for Mean Lifespan and Lifespan Disparity

If mortality develops regularly without any trend changes in the forecast years, the predictions of all three approaches appear to be close to the observed values. In Italy, we detect a regular increase in the average lifespan as well as a regular decline in lifespan disparity in the entire 1965–2009 period. Hence, Italian women experienced no trend changes, and their additional years of life were probably due to a compression of mortality that lasted (without any interruptions) in the reference and forecast years. Given these regular trends, the forecasts of all the approaches capture mean lifespan and its disparity with only negligible deviations. However, the MAPEs are smaller for *e*_0_ (0.3 % to 0.5 %) than for ${e}_0^{\dagger }$ (1.9 % for the Lee-Carter model and 1.5 % for the other two models).

#### Case of Japan: Regular Trend for Mean Lifespan and Irregular Trend for Lifespan Disparity

If the average lifespan trend is regular but the lifespan disparity trend is not, differences in the predictive ability of the three approaches are present but become visible only if we complement the evaluation with a measure of dispersion. In Japan, we observe a strong increase in the average lifespan from 1965 to 2009 as well as a decline in lifespan disparity that levels off in the forecast years. Hence, among Japanese women there was a trend change in the forecast years, and their additional years of life were probably due to a compression of mortality in the reference period and a shifting of mortality in the forecast years. Given the partial instability of mortality trends among Japanese women, the forecasts of the three models are close to the observed mean lifespan. The MAPEs for *e*_0_ range between 0.2 % and 0.3 %, suggesting that the forecasts were precise. However, the analysis of lifespan disparity shows that all the approaches overestimate the observed decline in the variability of the age at death. The deviations are greater for the Lee-Carter models (MAPEs for ${e}_0^{\dagger }$ are 8.7 % for the original model and 8.0 % for its rotated variant) than for the Bohk and Rau model (MAPE for ${e}_0^{\dagger }$ is 3.4 %). As a consequence, all three approaches predict a continuation of mortality compression while assuming that the concentration of deaths at higher ages will be greater than it actually was.

#### Case of Denmark: Irregular Trends for Mean Lifespan and Lifespan Disparity

If the trends of the mean lifespan and lifespan disparity are irregular, both evaluation measures indicate forecast errors. In Denmark, we observe an increase in the average lifespan in the forecast years after a period of stagnation in the 1980s and the early 1990s. We also observe a slight increase in lifespan disparity in the reference years that turns into a sharp decline in the forecast years. Hence, Danish women experienced trend changes in the forecast years. Their additional years of life were probably due to a mixture of a shifting and a worsening of mortality at different ages in the reference years as well as mortality compression in the forecast years. This result indicates that since the early 1990s, the mortality trends of Danish women have been catching up to those of vanguard populations, such as women in Italy and in Japan. Given these unstable mortality trends among Danish women, the forecasts of the three models capture the increasing trend of the average lifespan quite well. The MAPEs for *e*_0_, 0.6 % to 0.8 %, are only slightly higher than for Italy and Japan. However, the situation is different for lifespan disparity: the Lee-Carter models (more so the original model than the rotating variant) predict an increase in the forecast years despite an actual decline. This outcome not only deviates substantially from the observed values yielding MAPEs for ${e}_0^{\dagger }$ of 6.5 % and 5.4 %, but it also appears to be rather implausible given the general negative correlation between rising life expectancy at birth and declining lifespan disparity (see Fig. 1). In contrast, the Bohk and Rau model appears to capture the changing trend in lifespan disparity in the forecast years quite well, resulting in a more plausible forecast with only small deviations from the realized values (a MAPE for ${e}_0^{\dagger }$ of only 0.9 %).

#### Model Comparison

The illustrative examples suggest that the Lee-Carter model is less flexible than the other two models. This shortcoming is particularly noticeable when we look at the changing mortality trends in the forecast years, especially among women in Japan and Denmark. By contrast, the rotating variant and the Bohk and Rau model appear to be more capable of adapting to changing trends because unlike the original Lee-Carter model, which assumes that the relative changes are time-invariant across ages, these models assume that survival improvements will change over time. Analyzing the forecast errors, the rotated Lee-Carter model appears to perform on average better than the other two models because its MAPEs for *e*_0_ and ${e}_0^{\dagger }$ are relatively small for all countries and validation settings. By contrast, the original Lee-Carter model often has the largest MAPEs; and although the Bohk and Rau model often has the smallest MAPEs for life expectancy at birth and lifespan dispersion, it also has a few upward outliers that reduce its overall forecasting performance.

#### Sensitivity of the Results to the Reference Period

To examine the sensitivity of the above results to the choice of the reference period, we look at mortality forecasts up to 2009 that rely on different reference periods: 1960–1985, 1955–1980, and 1950–1975. The analyses are shown in Figs. S2–S4 in Online Resource 1, and have the same color scheme as in Fig. 2. Although the key message of the results presented is not affected by changing the reference period, a comparative analysis helps to identify method-based differences.

The fits of life expectancy at birth and lifespan disparity basically appear to depend on the regularity of mortality trends and the ability of the approaches to capture them appropriately. Because Japanese and Danish women experienced irregular mortality developments, making precise forecasts for them is particularly challenging. Thus, the predictive ability of the approaches declines as the magnitude of the MAPEs increases. This effect appears to be greater for the Lee-Carter model than for the other two models, and it appears to be more pronounced in forecasts of lifespan disparity than of average lifespan. For example, the greatest overall MAPE (10.7 %) is for ${e}_0^{\dagger }$ in Japan from the Lee-Carter model, whereas the smallest overall MAPE (0.4 %) is for *e*_0_ in Italy from the Bohk and Rau model (Table 2). The greater forecast error for the Lee-Carter model is probably due to the extrapolation of average trends of the reference period. Hence, if the overall trend of lifespan disparity is decreasing in the reference period, the Lee-Carter model tends to predict a decreasing pattern as well, and vice versa. However, the structural breaks in Danish and Japanese lifespan disparity appear to be unexpected and are therefore difficult to capture in a forecast generated by any model that has not been designed for this specific task. If we look instead at the more regular mortality trends in Italy, for example, we can see that the Lee-Carter models tend to generate rather conservative forecasts of progress in the average lifespan; that is, they tend to systematically underestimate the observed trajectories and yield overall MAPEs for *e*_0_ of 1.1 %. By contrast, the forecasts of the Bohk and Rau model yield a smaller overall MAPE for *e*_0_ (0.4 %) than the forecasts of the other two models, and they sometimes systematically overestimate the additional years of life. Examining lifespan disparity reveals even more differences between the approaches, particularly between the two Lee-Carter models in the forecasts of Japanese female mortality. The rotating variant appears to capture the flattening decline of lifespan disparity in the forecast years much better than the original model, and thus substantially improves forecasting performance: the overall MAPE for ${e}_0^{\dagger }$ in Japan is substantially lower for the rotated Lee-Carter variant (8.6 %) than for the original model (10.7 %). Also clearly visible for lifespan dispersion in Japan is that the further in time the reference period is, the more forecasts of the rotating variant diverge from those of the original model and converge with those of the Bohk and Rau model. Given that the rotation starts when life expectancy exceeds 75 years and that Japanese women reached this point in the early 1970s, this finding is not really surprising. As a consequence, the forecast that relies on data from 1950 to 1975 is also the forecast in which the rotation has the largest effect on the results. This finding demonstrates the need for time-variant survival improvements in order to capture dynamic trends in the variability of the age at death. The remaining deviations from the real values indicate that refining (or developing) forecasting approaches may help to account for patterns in lifespan disparity, such as the compression, shifting, and expansion of mortality. However, we do not expect forecasting errors to be equal to 0 because they show more signs of overfitting than of high forecasting performance.

Also of note is that the predictive ability of forecasts that rely on data from 1950 to 1975 appears to be lower than that of forecasts based on more recent mortality trends. Because this effect can be seen for the average lifespan and also partly for lifespan disparity, we speculate that it may be attributable to the delayed onset of the old-age mortality decline in the 1970s, which was crucial for future mortality developments in all three populations. Hence, if a major driving trend of mortality in the forecast years is missing in the reference period, the forecasting performance may be substantially reduced.

If we restrict our analysis to ages above 65, the relation of errors (MAPEs) for remaining years of life and lifespan disparity reverses. An exception is Japan, which shows larger errors for lifespan disparity than for remaining years of life at age 65, but only in the validation settings 1 and 2. Most likely, the onset of old-age mortality decline (Kannisto 1994) causes the reversal in the error pattern. Analyzing the magnitude of errors across all four validation settings provides evidence that the more years that are included in the reference period since the onset of the old-age mortality decline, the more accurate are the forecasts of remaining life expectancy at age 65. Given that the survival improvements at older ages primarily induced a parallel downward shift of the force of mortality on the log scale, the effects were large for *e*_65_ but only marginal for ${e}_{65}^{\dagger }$. This development is widely known as *shifting mortality* and has been described in detail by, for example, Bongaarts (2005) and Canudas-Romo (2008). Japan is the world record leader in terms of life expectancy thanks to exceptionally large old-age mortality improvements; we assume that these deviant/special trends in mortality may have caused larger changes in the variability at death that have not been captured in the forecasts and thus lead to larger errors for ${e}_{65}^{\dagger }$ in the last two validation settings (Cheung and Robine 2007; Wilmoth and Robine 2003).

## Summary and Concluding Remarks

Our analysis has shown that some methods—among them, the original Lee-Carter model, which is considered a golden standard in mortality forecasting—struggle to account for trends in lifespan disparity. This shortcoming, often caused by rather time-invariant survival improvements, has not been shown so clearly yet because the toolkit for evaluating the forecast performance focused on, for example, life expectancy at birth and age-specific death rates. These measures of *central tendency* are typically used to analyze *ex post* to what extent forecasts deviate from their realized values. Although these parameters of central tendency are useful for assessing how precisely average mortality has been forecasted, they cannot be used to determine whether the forecasted underlying mortality developments are plausible. This may be a serious drawback because similar average lifespans can result from different underlying mortality developments, which describe a dynamic age shift of survival improvement from younger to older ages in many highly developed countries in the last decades. As a consequence, small forecast errors of average lifespan do not necessarily indicate plausible trends in the forecasted underlying mortality dynamics. To assess whether the forecasts of the underlying developments are also plausible, we propose to use measures of lifespan disparity in the evaluation procedure. Despite many suitable measures of lifespan dispersion, we employed ${e}_0^{\dagger }$ as a measure of *spread* to tackle this problem.

In illustrative mortality forecasts for women in Italy, Japan, and Denmark—three populations who differ substantially in terms of lifespan disparity (see Fig. 1)—${e}_0^{\dagger }$ was a useful addition to the common toolkit for evaluating the predictive ability of forecasting approaches. We used the original Lee-Carter model (Lee and Carter 1992), its rotating variant proposed by Li et al. (2013), and the model of Bohk-Ewald and Rau (2017) to predict mortality up to 2009. Because the three approaches differ primarily in their ability to capture dynamic age shifts in the distribution of deaths, they are particularly suitable for evaluating how well they are able to forecast actual developments in average lifespan and lifespan disparity. To examine the sensitivity of our results, we chose four reference periods instead of just one: 1965–1990, 1960–1985, 1955–1980, and 1950–1970. We then compared the forecasted values of the average lifespan and lifespan disparity with the actual values.

The comparative analysis revealed that irrespective of the reference period, forecasting performance basically depends on the regularity (or continuation) of mortality trends and the ability of the approaches to capture them appropriately. Although the forecasts of life expectancy at birth generated by the Lee-Carter models are rather conservative, the forecasts generated by the Bohk and Rau model often have small forecast errors but also a few upward outliers. Moreover, the Japanese forecasts were found to be precise when we looked at average lifespan only, but they turned out to be rather inaccurate when we took lifespan disparity into account as well. Hence, the models were not able to capture the flattening decline of Japanese lifespan disparity in the forecast years, although the rotating model and the Bohk and Rau model fared better than the original Lee-Carter model because of time-variant survival improvements.

However, the remaining deviations from the observed values indicate that the refinement or the development of forecasting approaches should focus not only on average mortality but also on lifespan disparity. This indication may be particularly important given the concentration of mortality improvement potentials at the highest ages. Improving mortality at those ages could imply that people will probably live beyond current maximum ages. Hence, it will be crucial for forecasting approaches to be able to capture multiple trends in the (right) tail of the lifespan distribution (stagnation or expansion). As a consequence, the approaches should be able to forecast further reductions in mortality not only up to the current maximum ages but also to higher ages beyond. Doing so requires a high degree of modeling flexibility, which has been missing in existing approaches.

To summarize, our analysis illustrates that the joint evaluation of the average lifespan (*e*_0_) and the life years lost (${e}_0^{\dagger }$) provide new insights that we believe are needed for a comprehensive evaluation of the predictive performance of mortality forecasts. We also suggest that these new insights should be used when improving or developing new methods for forecasting mortality. Until now, these approaches were exclusively designed to capture the almost linear increase in life expectancy at birth. Hence, it is not surprising that forecasts of the average lifespan turn out to be more accurate and yield smaller forecast errors. The incorporation of lifespan disparity as a quality criterion or even central outcome may substantially improve the methodology.

## Electronic supplementary material

ESM 1 (PDF 332 kb)

## Footnotes

1 We use the terms “lifespan disparity,” “lifespan dispersion,” and “lifespan variability” interchangeably. Some scholars also call ${e}_0^{\dagger }$ “life disparity” (Vaupel et al. 2011). However, we use the term “lifespan disparity” to describe the general concept of lifespan variability that we measure using average life years lost.
