# Supplementary material for: Lifespan Disparity as an Additional Indicator for Evaluating Mortality Forecasts
Source: Demography. 2017 Jul 5;54(4):1559–77. doi: 10.1007/s13524-017-0584-0 (PMC5547182; doi:10.1007/s13524-017-0584-0)
Supplement: Supplementary file 1 — (PDF 332 kb) [file 13524_2017_584_MOESM1_ESM.pdf]

# Online Resource 1

## Lifespan Disparity as an Additional Indicator for Evaluating Mortality Forecasts

Christina Bohk-Ewald, Marcus Ebeling, and Roland Rau

# A Extending the age range beyond 110+

The data used are death counts and exposures by single age, 0 to 110+, from the Human Mortality Database (2015). To enable forecasting approaches to shift deaths to ages beyond 110+, we extend the age range of mortality data, like Ševčíková et al. (2016), with the model of Kannisto:

$$\mu_x \quad = \quad \frac{\alpha e^{\beta x}}{1 + \alpha e^{\beta x}} \tag{2}$$

as it is described in Thatcher et al. (1998, p. 16). We fit the model with the function *optim* in *R* (2015) to mortality at ages 80 to 110 using a Poisson log-likelihood for women in Italy, Denmark, and Japan for each year between 1950 and 2009. We then use the fitted Kannisto models to smooth mortality for ages 80 to 110, and to predict mortality for the ages above 110 in each year. Figure S1 depicts such mortality data for ages 80 to 130 for Italian, Danish, and Japanese women in the year 1950. The model of Kannisto is particularly suitable for populations with low mortality. It is also applied by, e.g., the Human Mortality Database and the United Nations (2014) to fit old-age mortality.

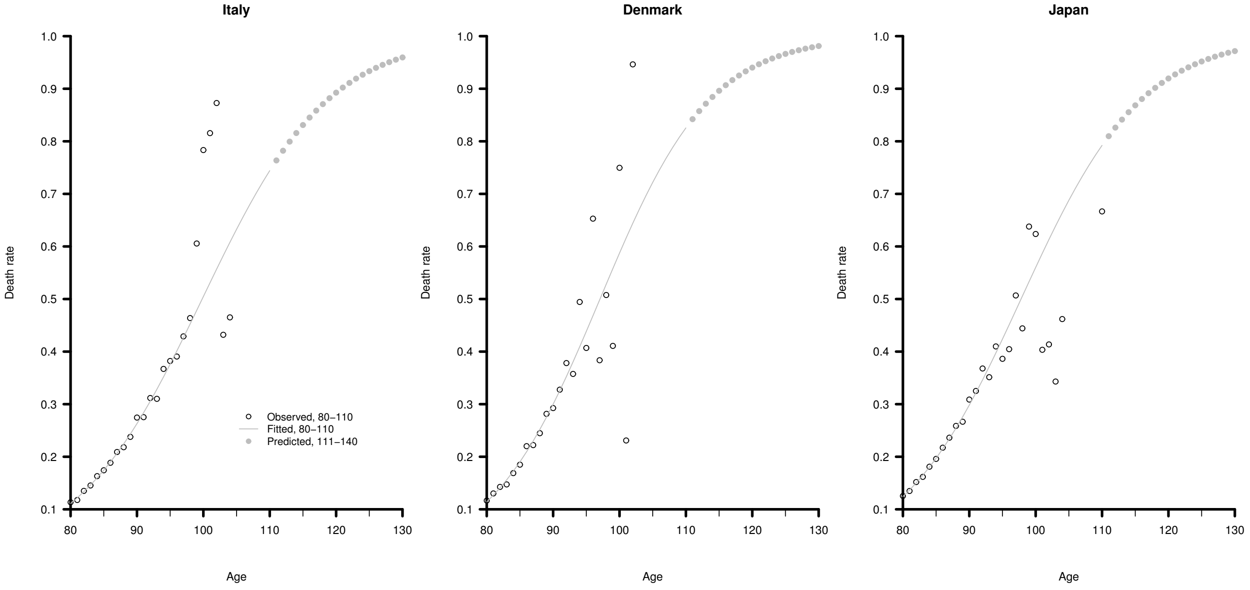


**Fig. S1** Extended age range of mortality data of Italian (left), Danish (center), and Japanese (right) women in 1950. We used the model of Kannisto to fit (gray line) observed mortality (black circles) at ages 80 to 110, and to predict it at ages 111 to 130 (gray dots).

## B Forecasts until 2009 with reference periods 1960-1985, 1955-1980, and 1950-1975

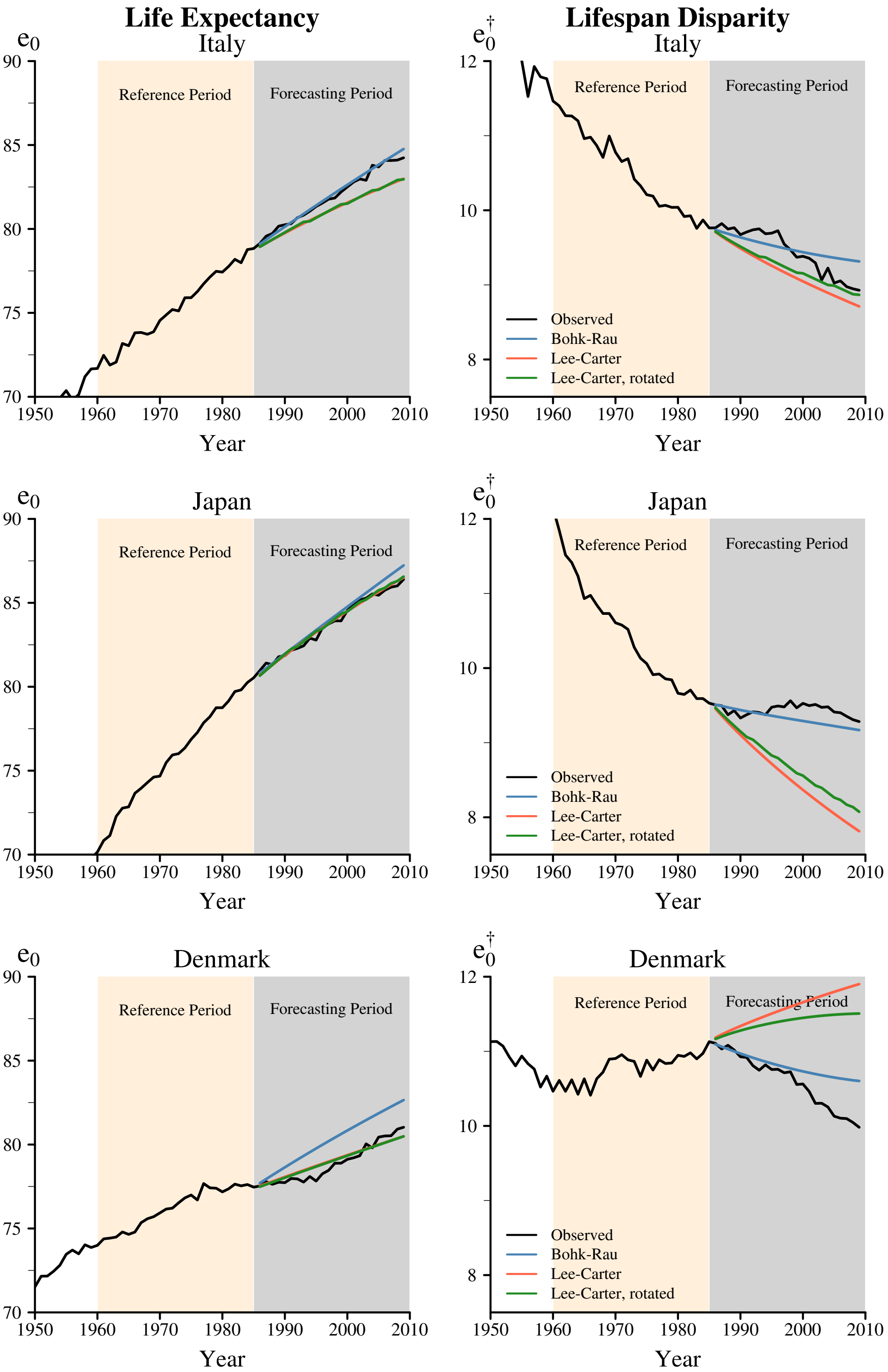


**Fig. S2** Life expectancy at birth (left panels) and life years lost at birth (right panels) for women in Italy (top), Japan (center), and Denmark (bottom); observed data are in black, forecasted data are in red (Lee-Carter model), green (rotating variant), and blue (Bohk-Rau model); reference period: 1960-1985.

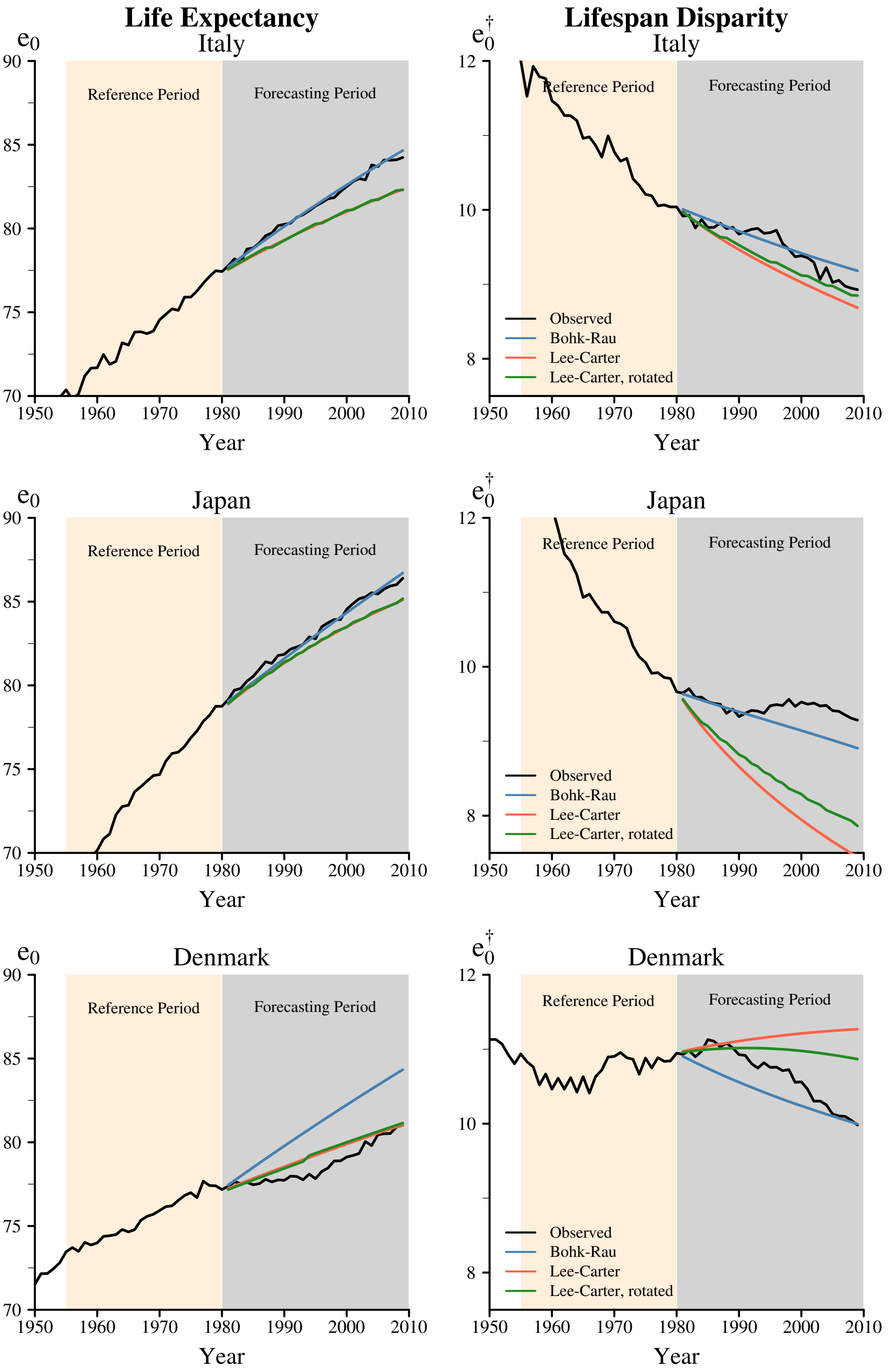


**Fig. S3** Life expectancy at birth (left panels) and life years lost at birth (right panels) for women in Italy (top), Japan (center), and Denmark (bottom); observed data are in black, forecasted data are in red (Lee-Carter model), green (rotating variant), and blue (Bohk-Rau model); reference period: 1955-1980.

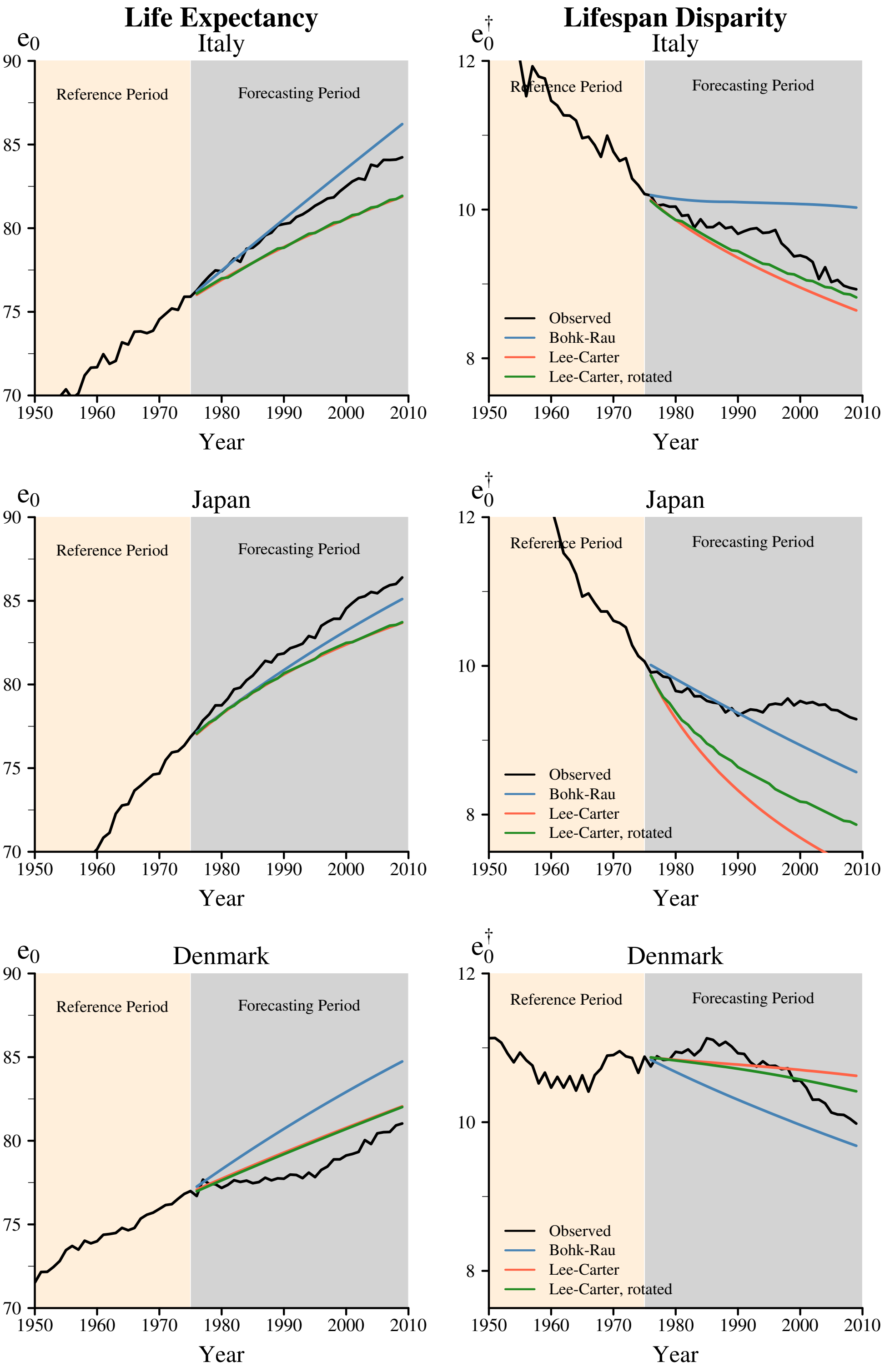


**Fig. S4** Life expectancy at birth (left panels) and life years lost at birth (right panels) for women in Italy (top), Japan (center), and Denmark (bottom); observed data are in black, forecasted data are in red (Lee-Carter model), green (rotating variant), and blue (Bohk-Rau model); reference period: 1950-1975.

## C MAPE estimates for $e_{65}$ and $e^{\dagger}_{65}$

| Country | Measure | LC | LC, rotated | Bohk–Rau |
|---|---|---|---|---|
| **Validation 1** (Ref.years: 1965–1990; Forecast years: 1991–2009) | | | | |
| Italy | $e_{65}$ | 0.016 | 0.013 | 0.014 |
| | $e^{\dagger}_{65}$ | 0.009 | 0.008 | 0.021 |
| Japan | $e_{65}$ | 0.015 | 0.012 | 0.008 |
| | $e^{\dagger}_{65}$ | 0.056 | 0.050 | 0.012 |
| Denmark | $e_{65}$ | 0.020 | 0.018 | 0.026 |
| | $e^{\dagger}_{65}$ | 0.016 | 0.013 | 0.010 |
| **Validation 2** (Ref.years: 1960–1985; Forecast years: 1986–2009) | | | | |
| Italy | $e_{65}$ | 0.046 | 0.040 | 0.008 |
| | $e^{\dagger}_{65}$ | 0.016 | 0.012 | 0.034 |
| Japan | $e_{65}$ | 0.025 | 0.018 | 0.007 |
| | $e^{\dagger}_{65}$ | 0.050 | 0.041 | 0.020 |
| Denmark | $e_{65}$ | 0.054 | 0.046 | 0.062 |
| | $e^{\dagger}_{65}$ | 0.051 | 0.045 | 0.044 |
| **Validation 3** (Ref.years: 1955–1980; Forecast years: 1981–2009) | | | | |
| Italy | $e_{65}$ | 0.055 | 0.049 | 0.006 |
| | $e^{\dagger}_{65}$ | 0.027 | 0.022 | 0.020 |
| Japan | $e_{65}$ | 0.068 | 0.056 | 0.018 |
| | $e^{\dagger}_{65}$ | 0.067 | 0.055 | 0.008 |
| Denmark | $e_{65}$ | 0.063 | 0.057 | 0.099 |
| | $e^{\dagger}_{65}$ | 0.009 | 0.010 | 0.012 |
| **Validation 4** (Ref.years: 1950–1975; Forecast years: 1976–2009) | | | | |
| Italy | $e_{65}$ | 0.065 | 0.057 | 0.041 |
| | $e^{\dagger}_{65}$ | 0.026 | 0.020 | 0.076 |
| Japan | $e_{65}$ | 0.105 | 0.085 | 0.050 |
| | $e^{\dagger}_{65}$ | 0.071 | 0.054 | 0.029 |
| Denmark | $e_{65}$ | 0.066 | 0.060 | 0.102 |
| | $e^{\dagger}_{65}$ | 0.011 | 0.011 | 0.009 |

**Table S1** Mean of the Absolute Percentage Errors (MAPE) for $e_{65}$ and $e^{\dagger}_{65}$ over the forecast years by country and method. MAPEs are shown for four validating settings that all forecast mortality until 2009, but use different historical periods.

| Country | Measure | LC | LC, rotated | Bohk-Rau |
|---|---|---|---|---|
| **Average across all validation settings** | | | | |
| Italy | $e_{65}$ | 0.046 | 0.040 | 0.017 |
| | $e^{\dagger}_{65}$ | 0.020 | 0.016 | 0.038 |
| Japan | $e_{65}$ | 0.053 | 0.043 | 0.021 |
| | $e^{\dagger}_{65}$ | 0.061 | 0.050 | 0.017 |
| Denmark | $e_{65}$ | 0.051 | 0.045 | 0.072 |
| | $e^{\dagger}_{65}$ | 0.021 | 0.019 | 0.019 |

**Table S2** Mean of the Absolute Percentage Errors (MAPE) for $e_{65}$ and $e^{\dagger}_{65}$ over all validation settings by country and method.

# D Empirical frequencies for $e_0$ and $e_0^\dagger$

| Country | Measure | LC | LC, rotated | Bohk–Rau |
|---|---|---|---|---|
| **Validaton 1** (Ref.years: 1965–1990; Forecast years: 1991–2009) | | | | |
| Italy | $e_0$ | 100.00 | 100.00 | 94.74 |
| | $e_0^\dagger$ | 10.53 | 31.58 | 36.84 |
| Japan | $e_0$ | 100.00 | 100.00 | 94.74 |
| | $e_0^\dagger$ | 0.00 | 0.00 | 0.00 |
| Denmark | $e_0$ | 100.00 | 100.00 | 89.47 |
| | $e_0^\dagger$ | 0.00 | 0.00 | 42.11 |
| **Validation 2** (Ref.years: 1960–1985; Forecast years: 1986–2009) | | | | |
| Italy | $e_0$ | 79.17 | 91.67 | 91.67 |
| | $e_0^\dagger$ | 0.00 | 20.83 | 41.67 |
| Japan | $e_0$ | 100.00 | 100.00 | 87.50 |
| | $e_0^\dagger$ | 0.00 | 0.00 | 50.00 |
| Denmark | $e_0$ | 100.00 | 100.00 | 54.17 |
| | $e_0^\dagger$ | 0.00 | 0.00 | 37.50 |
| **Validation 3** (Ref.years: 1955–1980; Forecast years: 1981–2009) | | | | |
| Italy | $e_0$ | 55.17 | 72.41 | 89.66 |
| | $e_0^\dagger$ | 13.79 | 34.48 | 48.28 |
| Japan | $e_0$ | 100.00 | 100.00 | 48.28 |
| | $e_0^\dagger$ | 0.00 | 0.00 | 96.55 |
| Denmark | $e_0$ | 100.00 | 100.00 | 55.17 |
| | $e_0^\dagger$ | 13.79 | 13.79 | 37.93 |
| **Validation 4** (Ref.years: 1950–1975; Forecast years: 1976–2009) | | | | |
| Italy | $e_0$ | 64.71 | 79.41 | 88.24 |
| | $e_0^\dagger$ | 5.88 | 26.47 | 2.94 |
| Japan | $e_0$ | 64.71 | 100.00 | 26.47 |
| | $e_0^\dagger$ | 2.94 | 2.94 | 82.35 |
| Denmark | $e_0$ | 100.00 | 100.00 | 41.18 |
| | $e_0^\dagger$ | 20.59 | 17.65 | 32.35 |

**Table S3** Empirical frequencies, in %, for the 95% prediction intervals of $e_0$ and $e_0^\dagger$ over the forecast years by country and method. The empirical frequencies are shown for four validating settings that all forecast mortality until 2009, but use different historical periods.

## E Empirical frequencies for $e_{65}$ and $e^{\dagger}_{65}$

| Country | Measure | LC | LC, rotated | Bohk–Rau |
|---|---|---|---|---|
| **Validaton 1** (Ref.years: 1965–1990; Forecast years: 1991–2009) | | | | |
| Italy | $e_{65}$ | 94.74 | 100.00 | 100.00 |
| | $e^{\dagger}_{65}$ | 21.05 | 10.53 | 57.89 |
| Japan | $e_{65}$ | 100.00 | 100.00 | 47.37 |
| | $e^{\dagger}_{65}$ | 0.00 | 0.00 | 63.16 |
| Denmark | $e_{65}$ | 100.00 | 100.00 | 57.89 |
| | $e^{\dagger}_{65}$ | 31.58 | 36.84 | 73.68 |
| **Validation 2** (Ref.years: 1960–1985; Forecast years: 1986–2009) | | | | |
| Italy | $e_{65}$ | 4.17 | 29.17 | 83.33 |
| | $e^{\dagger}_{65}$ | 4.17 | 12.50 | 66.67 |
| Japan | $e_{65}$ | 70.83 | 95.83 | 50.00 |
| | $e^{\dagger}_{65}$ | 4.17 | 4.17 | 91.67 |
| Denmark | $e_{65}$ | 100.00 | 100.00 | 45.83 |
| | $e^{\dagger}_{65}$ | 0.00 | 0.00 | 8.33 |
| **Validation 3** (Ref.years: 1955–1980; Forecast years: 1981–2009) | | | | |
| Italy | $e_{65}$ | 13.79 | 20.69 | 93.10 |
| | $e^{\dagger}_{65}$ | 0.00 | 0.00 | 75.86 |
| Japan | $e_{65}$ | 6.90 | 41.38 | 24.14 |
| | $e^{\dagger}_{65}$ | 0.00 | 0.00 | 93.10 |
| Denmark | $e_{65}$ | 100.00 | 100.00 | 55.17 |
| | $e^{\dagger}_{65}$ | 48.28 | 41.38 | 89.66 |
| **Validation 4** (Ref.years: 1950–1975; Forecast years: 1976–2009) | | | | |
| Italy | $e_{65}$ | 20.59 | 35.29 | 94.12 |
| | $e^{\dagger}_{65}$ | 2.94 | 2.94 | 61.76 |
| Japan | $e_{65}$ | 5.88 | 38.24 | 23.53 |
| | $e^{\dagger}_{65}$ | 2.94 | 0.00 | 94.12 |
| Denmark | $e_{65}$ | 100.00 | 100.00 | 61.76 |
| | $e^{\dagger}_{65}$ | 35.29 | 41.18 | 88.24 |

**Table S4** Empirical frequencies, in %, for the 95% prediction intervals of $e_{65}$ and $e^{\dagger}_{65}$ over the forecast years by country and method. The empirical frequencies are shown for four validating settings that all forecast mortality until 2009, but use different historical periods.
